# Enterotype-Specific Effect of Human Gut Microbiota on the Fermentation of Marine Algae Oligosaccharides: A Preliminary Proof-of-Concept In Vitro Study

**DOI:** 10.3390/polym14040770

**Published:** 2022-02-16

**Authors:** Tianyu Fu, Luning Zhou, Zhiliang Fu, Bin Zhang, Quancai Li, Lin Pan, Chen Zhou, Qing Zhao, Qingsen Shang, Guangli Yu

**Affiliations:** 1 Key Laboratory of Marine Drugs of Ministry of Education, Shandong Provincial Key Laboratory of Glycoscience and Glycotechnology, School of Medicine and Pharmacy, Ocean University of China, Qingdao 266003, China; futianyufty413@163.com (T.F.); huahai@ouc.edu.cn (L.Z.); fuzhiliang@ouc.edu.cn (Z.F.); zhangbin0993@sina.com (B.Z.); quancaili@126.com (Q.L.); pl_panlin@163.com (L.P.); zhou_chen2000@126.com (C.Z.); zq010508@163.com (Q.Z.); 2 Qingdao Marine Biomedical Research Institute, Qingdao 266071, China; 3 Laboratory for Marine Drugs and Bioproducts, Qingdao National Laboratory for Marine Science and Technology, Qingdao 266003, China

**Keywords:** enterotype, gut microbiota, marine algae oligosaccharides, alginate oligosaccharides, agar oligosaccharides, carrageenan oligosaccharides, fermentation, prebiotics

## Abstract

The human gut microbiota plays a critical role in the metabolism of dietary carbohydrates. Previous studies have illustrated that marine algae oligosaccharides could be utilized and readily fermented by human gut microbiota. However, the human gut microbiota is classified into three different enterotypes, and how this may affect the fermentation processes of marine algae oligosaccharides has not been studied. Here, using in vitro fermentation and 16 S high-throughput sequencing techniques, we demonstrate that the human gut microbiota has an enterotype-specific effect on the fermentation outcomes of marine algae oligosaccharides. Notably, microbiota with a *Bacteroides* enterotype was more proficient at fermenting carrageenan oligosaccharides (KOS) as compared to that with a *Prevotella* enterotype and that with an *Escherichia* enterotype. Interestingly, the prebiotic effects of marine algae oligosaccharides were also found to be enterotype dependent. Altogether, our study demonstrates an enterotype-specific effect of human gut microbiota on the fermentation of marine algae oligosaccharides. However, due to the availability of the fecal samples, only one sample was used to represent each enterotype. Therefore, our research is a proof-of-concept study, and we anticipate that more detailed studies with larger sample sizes could be conducted to further explore the enterotype-specific prebiotic effects of marine oligosaccharides.

## 1. Introduction

The human gut microbiota, which is composed of more than 1000 bacterial species, is a highly complex microbial community [1,2,3]. It is estimated that the human gut microbiota has a total gene set of about 3 million, which is about 150 times larger than that of the host genome [4,5]. With such a great number of genes, the gut microbiota contributes significantly to the metabolism of the dietary nutrients, including carbohydrates, proteins, vitamins, and fats [6,7,8]. Marine algae oligosaccharides are a class of functional oligosaccharides that are derived from alginate, agar, and carrageenan. Previous studies have indicated that marine algae oligosaccharides could be easily fermented and metabolized by human gut microbiota [9,10]. Using an in vitro fermentation model, we demonstrated that *Bacteroides xylanisolvens*, *Bacteroides ovatus*, and *Bacteroides uniforms* are three major degraders for marine algae oligosaccharides in the colon [9,10]. Fermentation of marine algae oligosaccharides by the gut microbiota could produce a significant amount of short-chain fatty acids and also change the structure of the microbiome [9,10]. Recently, Han et al. investigated the effect of marine algae oligosaccharides on the pig gut microbiota and found that they could stimulate the growth of beneficial microbes including *Roseburia* spp. and *Faecalibacterium* spp. [11]. The potential beneficial effects of marine algae oligosaccharides on the gut microbiota make them good candidates for the development of next-generation prebiotics [9,11]. However, although interesting results have been found for pigs, what effects the marine algae oligosaccharides have on the human gut microbiota have not been studied.

In 2011, Peer Bork and colleagues illustrated that the human gut microbiota is classified into three different enterotypes [12]. Different enterotypes of human gut microbiota were characterized with different species of gut microbes [12,13]. Recently, we illustrated that the enterotype could pose a significant effect on the fermentation outcomes of dietary fibers, including alginate and its derivatives [14]. Bacteroides-dominated microbiota is more proficient at fermenting dietary polysaccharides as compared to *Prevotella*-dominated microbiota and *Escherichia*-dominated microbiota. Fermentation of alginate and its derivative polymannuronate acid by *Bacteroides*-dominated microbiota produced the highest amount of total short-chain fatty acids and butyrate [14]. The enterotype opens a new window to understand the metabolic functions of the human gut microbiota and has gained tremendous attention in both academic and industrial sectors [15,16]. However, how the enterotype may affect the fermentation processes of marine algae oligosaccharides by the human gut microbiota has not been explored.

In the present study, we aimed to investigate what effects the marine algae oligosaccharides have on the human gut microbiota and how the enterotype may affect the fermentation outcomes of these functional oligosaccharides. We hope that the current study will bring new insights to understand the potential prebiotic effects and the metabolic processes of marine algae oligosaccharides by the human gut microbiota.

## 2. Results

### 2.1. Fermentation and Utilization Profiles of Marine Algae Oligosaccharides

We tested the enterotype of 30 healthy volunteers but, unfortunately, we only found one *Escherichia* enterotype microbiota. Therefore, in the present study, only one sample was used to represent each enterotype. Subject F1 was identified to be an *Escherichia* enterotype microbiota. Subject F17 was identified to be a *Bacteroides* enterotype microbiota. Subject F18 was identified to be a *Prevotella* enterotype microbiota. The bacterial compositions of the three enterotypes of gut microbiota are shown in Figures S1 and S2. The data are a reanalysis of previous samples published by our lab [14]. F17 and F18 were used because they could best illustrate the different characteristics of the two enterotypes.

**Figure 1:**
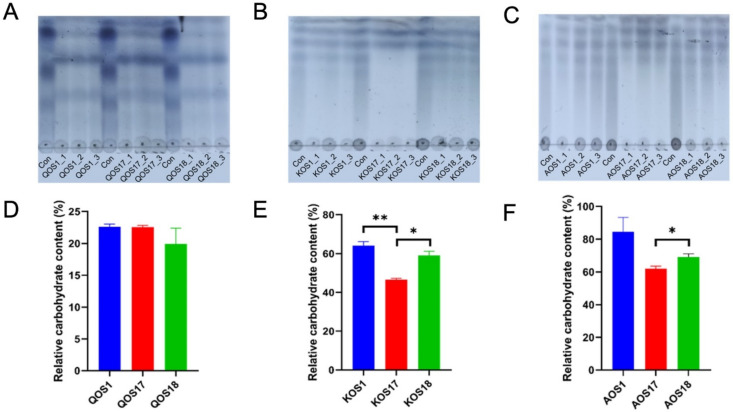
Fermentation and utilization of QOS, KOS, and AOS by three different enterotypes of human microbiota. F1 was identified to be an *Escherichia* enterotype microbiota, F17 was identified to be a *Bacteroides* enterotype microbiota, and F18 was identified to be a *Prevotella* enterotype microbiota. QOS1 denotes fermentation of QOS by gut microbiota F1. QOS17 denotes fermentation of QOS by gut microbiota F17. QOS18 denotes fermentation of QOS by gut microbiota F18. Each fermentation group has three replicates. KOS and AOS were labeled using the same format. TLC analysis of the oligosaccharides before and after fermentation (**A**–**C**). Con is the control medium at the beginning of fermentation. Utilization of the oligosaccharides during fermentation (**D**–**F**). All the fermentation experiments were performed in triplicates. Data are expressed as mean ± SEM. Statistical analysis was performed using ANOVA with post hoc Tukey’s multiple comparisons test. * *p* < 0.05, ** *p* < 0.01.

Thin-layer chromatography (TLC) and total carbohydrate analysis indicated that the enterotype of the gut microbiota could affect the utilization profiles of marine algae oligosaccharides (Figure 1B–G). Specifically, microbiota with a *Bacteroides* enterotype was more proficient at fermenting carrageenan oligosaccharides (KOS) as compared to that with a *Prevotella* enterotype and that with an *Escherichia* enterotype (Figure 1C,F). Interestingly, a similar pattern was also seen for alginate oligosaccharides (AOS) (Figure 1D,G). However, it is of significance to note that the three enterotypes of gut microbiota have indistinguishable effects on the utilization profiles of agar oligosaccharides (QOS) (Figure 1B,E). QOS, AOS, and KOS are structurally different (Figure S2), and this indicated that the chemical structures could also affect the fermentation processes of the marine algae oligosaccharides.

### 2.2. Diversity Analysis of the Gut Microbiota

**Figure 2:**
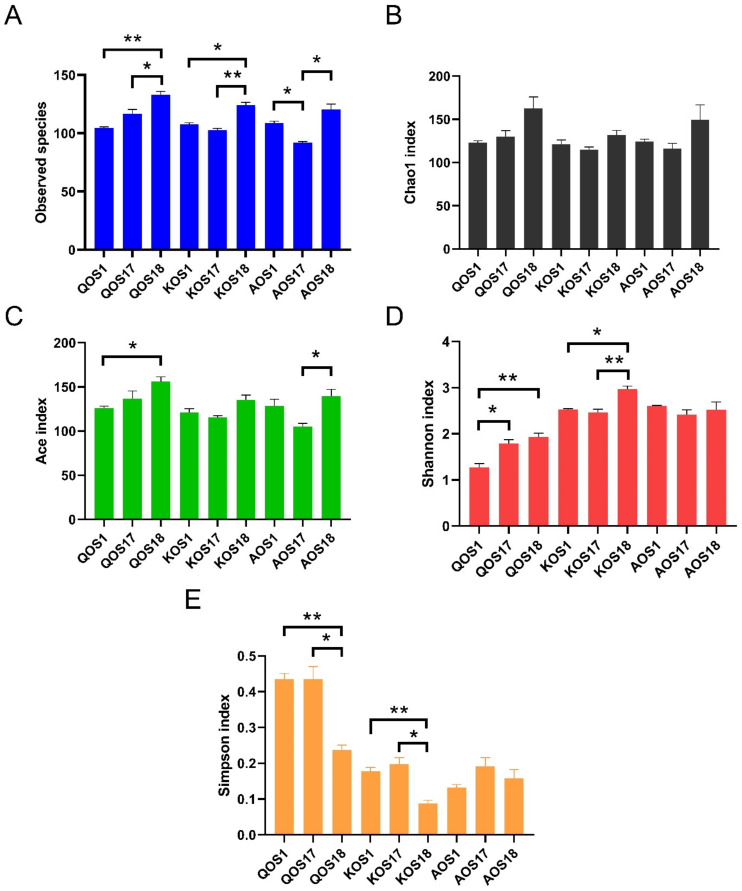
The α−diversity analysis of the gut microbiota. Observed species (**A**), Chao1 index (**B**), Ace index (**C**), Shannon index (**D**), and Simpson index (**E**). QOS1 denotes fermentation of QOS by gut microbiota F1. QOS17 denotes fermentation of QOS by gut microbiota F17. QOS18 denotes fermentation of QOS by gut microbiota F18. Each fermentation group has three replicates. KOS and AOS were labeled using the same format. Data are expressed as mean ± SEM. Statistical analysis was performed using ANOVA with post hoc Tukey’s multiple comparisons test. * *p* < 0.05, ** *p* < 0.01.

**Figure 3:**
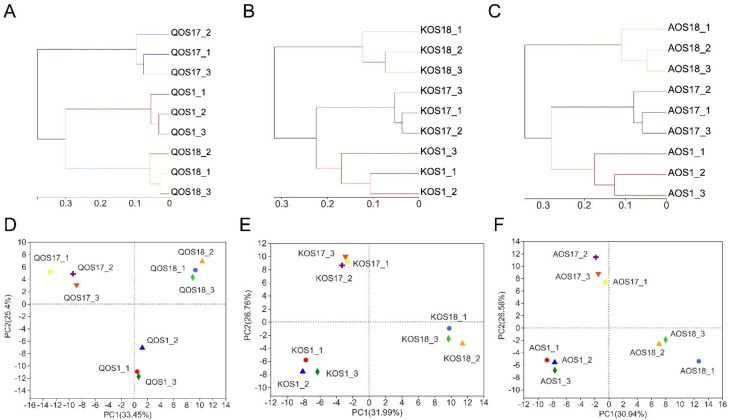
The *β*−diversity analysis of the gut microbiota. Clustering analysis of the gut microbiota (**A**–**C**). PCA score plot analysis of the gut microbiota (**D**–**F**). QOS1 denotes fermentation of QOS by gut microbiota F1. QOS17 denotes fermentation of QOS by gut microbiota F17. QOS18 denotes fermentation of QOS by gut microbiota F18. Each fermentation group has three replicates. KOS and AOS were labeled using the same format.

To understand the effect of enterotype on the fermentation processes of marine algae oligosaccharides, we further analyzed the composition of the gut microbiota using 16 S high-throughput sequencing. Bioinformatic analysis indicated that enterotype could affect both the *α*−diversity and *β*−diversity of the gut microbiota during fermentation (Figure 2 and Figure 3).

Specifically, *Prevotella* enterotype microbiota tended to include the largest amount of and most diversified bacterial species for the fermentation. In contrast, *Bacteroides* enterotype microbiota and *Escherichia* enterotype microbiota tended to include a smaller amount of and less diversified bacterial species for the fermentation (Figure 2A,D). Specifically, there was a clear separation of the three different enterotypes of gut microbiota by Clustering and PCA score plot analyses (Figure 3). This indicates that different enterotypes of the gut microbiota will drive the fermentation process towards different directions. Altogether, our results illustrated that the enterotype could pose a significant effect on the diversity of the gut microbiota during fermentation of marine algae oligosaccharides.

### 2.3. Compositional Analysis of the Gut Microbiota

**Figure 4:**
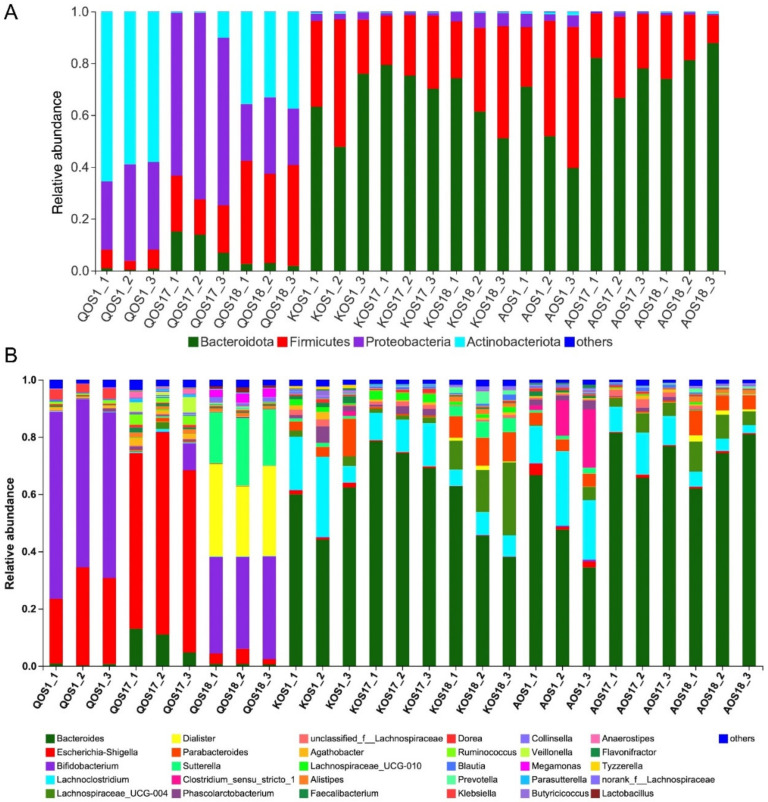
Compositional analysis of the gut microbiota. Microbiota composition at the phylum level (**A**) and genus level (**B**). QOS1 denotes fermentation of QOS by gut microbiota F1. QOS17 denotes fermentation of QOS by gut microbiota F17. QOS18 denotes fermentation of QOS by gut microbiota F18. Each fermentation group has three replicates. KOS and AOS were labeled using the same format.

To find out which bacterium was responsible for the fermentation and utilization of the marine algae oligosaccharides, we then analyzed the composition of the gut microbiota at the phylum level (Figure 4A) and genus level (Figure 4B). As expected, different bacteria were found to be involved in the fermentation of the QOS, KOS, and AOS. Specifically, KOS and AOS were fermented primarily by *Bacteroides* spp. whereas QOS were fermented mostly by *Bifidobacterium* spp. and *Escherichia-Shigella* spp. (Figure 4B).

**Figure 5:**
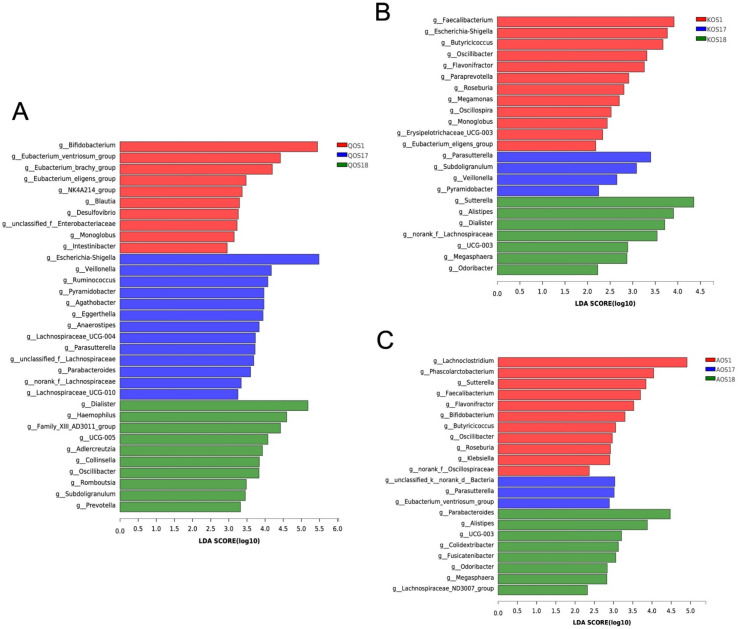
LEfSe LDA analysis of the microbiota during fermentation of QOS (**A**), KOS (**B**), and AOS (**C**). The analysis was performed at the genus level. Only taxa with an LDA score >2 are listed. QOS1 denotes fermentation of QOS by gut microbiota F1. QOS17 denotes fermentation of QOS by gut microbiota F17. QOS18 denotes fermentation of QOS by gut microbiota F18. Each fermentation group has three replicates. KOS and AOS were labeled using the same format. The LEfSe analysis was performed at the genus level. Only taxa with an LDA score >2 are listed.

To study the enterotype-specific effect of gut microbiota on the fermentation of marine algae oligosaccharides, we next conducted a linear discriminant analysis effect size (LEfSe) analysis (Figure 5). LEfSe analysis further confirmed that each enterotype of gut microbiota has its own unique bacteria that could metabolize and ferment marine algae oligosaccharides. For QOS, the major fermenters from *Escherichia* enterotype microbiota were *Bifidobacterium* spp. whereas those from *Bacteroides* enterotype microbiota and *Prevotella* enterotype microbiota were *Escherichia*-*Shigella* spp. and *Dialister* spp., respectively (Figure 5A). For KOS, the major fermenters from *Escherichia* enterotype microbiota were *Faecalibacterium* spp. whereas those from *Bacteroides* enterotype microbiota and *Prevotella* enterotype microbiota were *Parasutterella* spp. and *Sutterella* spp., respectively (Figure 5B). For QOS, the major fermenters from *Escherichia* enterotype microbiota were *Lachnoclostridium* spp. while those from *Bacteroides* enterotype microbiota and *Prevotella* enterotype microbiota were and *Eubacterium ventriosum* and *Parabacteroides* spp. (Figure 5C).

QOS, AOS, and KOS are composed of unique monosaccharides and have structurally different glycosidic linkage modes. Previous studies have indicated that different gut bacteria have different classes of carbohydrate-active enzymes (CAZymes) [17,18,19], and, since CAZymes are required for the metabolism of marine algae oligosaccharides [19,20], it is therefore reasonable that different enterotypes of human gut microbiota would have varying effects on the fermentation processes of QOS, KOS, and AOS.

### 2.4. Prebiotic Effects of Marine Algae Oligosaccharides

Previous studies have indicated that marine algae oligosaccharides, including QOS, KOS, and AOS, could promote the growth of beneficial microbes in the gut [9,11]. In the present study, we found that the effects of marine algae oligosaccharides on the gut microbiota are enterotype specific. In this light, we then wondered if enterotype could also affect the prebiotic effect of marine algae oligosaccharides.

**Figure 6:**
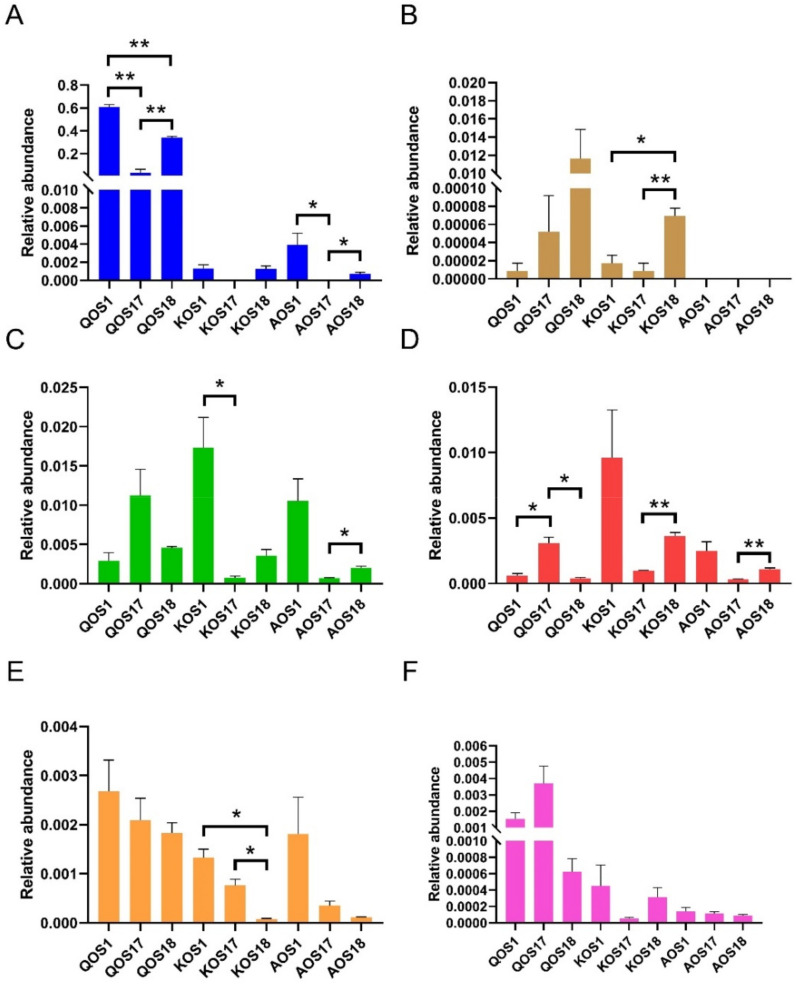
Comparison of the prebiotic effects of QOS, KOS, and AOS on the human gut microbiota. Relative abundances of *Bifidobacterium* spp. (**A**), *Lactobacillus* spp. (**B**), *Faecalibacterium* spp. (**C**), *Butyricicoccus* spp. (**D**), *Roseburia* spp. (**E**), and *Eubacterium hallii* (**F**). QOS1 denotes fermentation of QOS by gut microbiota F1. QOS17 denotes fermentation of QOS by gut microbiota F17. QOS18 denotes fermentation of QOS by gut microbiota F18. Each fermentation group has three replicates. KOS and AOS were labeled using the same format. Data are expressed as mean ± SEM. Statistical analysis was performed using ANOVA with post hoc Tukey’s multiple comparisons test. * *p* < 0.05, ** *p* < 0.01.

To answer this question, we further compared the relative abundances of probiotic bacteria in the three different enterotypes of gut microbiota. Interestingly, the prebiotic effects of QOS, KOS, and AOS were also found to be enterotype specific (Figure 6). For example, the bifidogenic effects of QOS and AOS were more prominent in *Prevotella* enterotype microbiota and *Escherichia* enterotype microbiota as compared to *Bacteroides* enterotype microbiota (Figure 6A). Similarly, the prebiotic effect of KOS on *Lactobacillus* spp. was also observed to be dependent on the enterotype (Figure 6B). Additionally, marine algae oligosaccharides were also noted to have enterotype-specific prebiotic effects on butyrate-producing bacteria, which are next-generation probiotics (Figure 6C–E).

Together, our results indicate that the prebiotic effects of QOS, KOS, and AOS might be enterotype specific, and caution should be given to individual’s composition of the gut microbiota when evaluating the prebiotic effect of marine algae oligosaccharides in human trials.

### 2.5. Fermentation Products Analysis

**Figure 7:**
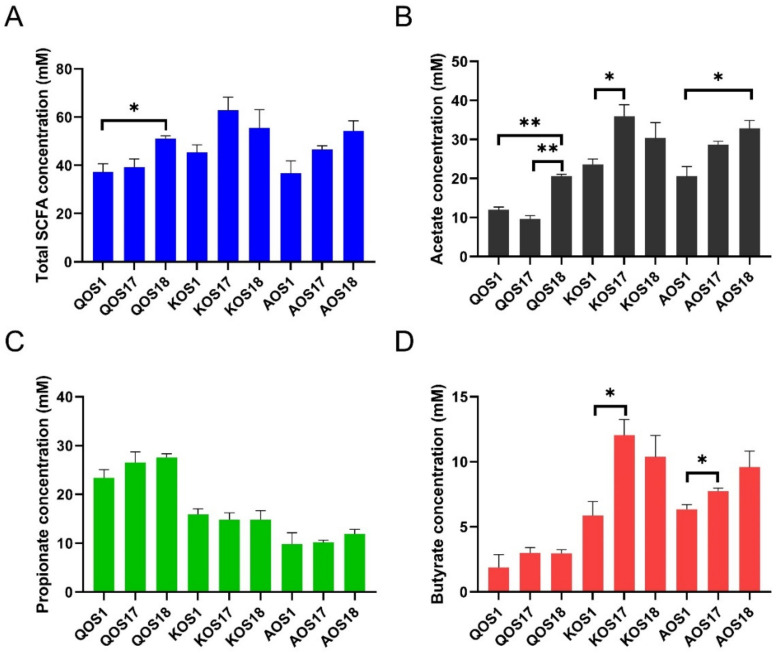
Production of short-chain fatty acid (SCFA) by three different enterotypes of human microbiota during fermentation of QOS, KOS, and AOS. The concentrations of total SCFA (**A**), acetate (**B**), propionate (**C**), and butyrate (**D**) were analyzed. QOS1 denotes fermentation of QOS by gut microbiota F1. QOS17 denotes fermentation of QOS by gut microbiota F17. QOS18 denotes fermentation of QOS by gut microbiota F18. Each fermentation group has three replicates. KOS and AOS were labeled using the same format. Data are expressed as mean ± SEM. Statistical analysis was performed using ANOVA with post hoc Tukey’s multiple comparisons test. * *p* < 0.05, ** *p* < 0.01.

Short-chain fatty acids (SCFAs), including acetate, propionate, and butyrate, are a class of major fermentation products of marine algae oligosaccharides [21,22,23]. Given that the effects of gut microbiota on the fermentations of QOS, KOS, and AOS are enterotype specific, we next wondered whether enterotype could also affect the production of SCFAs (Figure 7). Interestingly, enterotype was also found to have a significant effect on the production of SCFAs, especially acetate and butyrate (Figure 7B,D). Compared to that of *Escherichia* enterotype microbiota, *Bacteroides* enterotype microbiota and *Prevotella* enterotype microbiota produced a higher amount of acetate and butyrate during fermentation of QOS, KOS, and AOS.

## 3. Discussion

Preceding studies have also found a similar effect of enterotype on the production of SCFAs when fermenting alginate and its derivatives using human gut microbiota [14]. In the present study, we extend previous findings of the enterotype-specific effect of human gut microbiota from the fermentation of polysaccharides to that of prebiotic oligosaccharides. Our results could help to explain why the same prebiotic preparations have dissimilar effects among different individuals [24]. We anticipate that more studies will be conducted to further explore the enterotype-specific prebiotic effect of oligosaccharides.

The current study has one limitation. We tested the enterotype of 30 healthy volunteers but, unfortunately, we only found one *Escherichia* enterotype microbiota [14]. Therefore, in the present study, only one sample was used to represent each enterotype. The present research is a preliminary proof-of-concept study. We are still trying to find more volunteers to participate in the research. Our study provides a potential new way to understand the prebiotic effects of marine oligosaccharides. We anticipate that more detailed studies with larger sample sizes could be conducted to further explore the enterotype-specific prebiotic effects of marine oligosaccharides.

**Figure 8:**
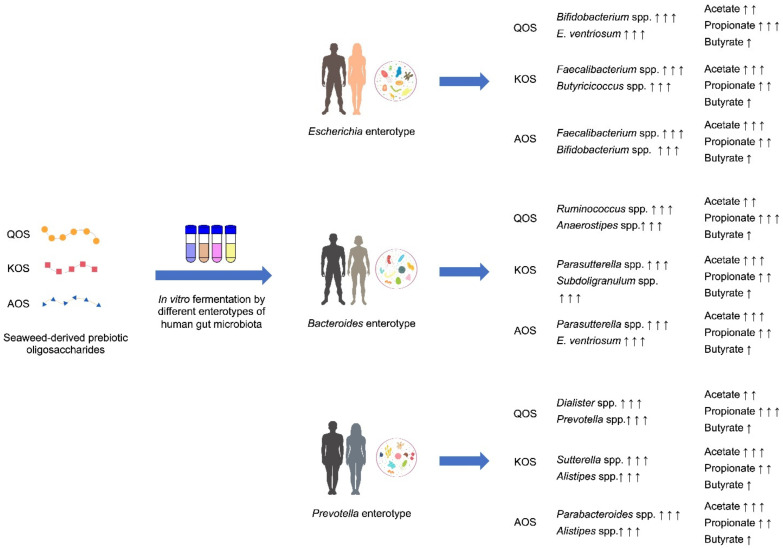
A graphical summary of the main findings of the present study.

Altogether, our study demonstrates an enterotype-specific effect of human gut microbiota on the fermentation of marine algae oligosaccharides (Figure 8). Enterotype could dictate the fermentation processes of QOS, KOS, and AOS and the proportions of SCFAs that are produced. Additionally, enterotype could also affect the prebiotic potential of marine algae oligosaccharides. Our study sheds new light onto the metabolism of QOS, KOS, and AOS by human gut microbiota and highlights that the use of prebiotics should be tailored in terms of the individual’s enterotype in the upcoming era of precision nutrition.

## 4. Materials and Methods

### 4.1. Chemicals and Reagents

Marine algae oligosaccharides, including QOS, KOS, and AOS, all with a molecular weight of about 2.0 kDa, were kindly provided by Qingdao International Oligose Preparation Center (Qingdao, China). The chemical structures of QOS, KOS, and AOS are shown in Figure S3. The other chemicals and reagents used in the present study were obtained from Sigma (Shanghai, China).

### 4.2. In Vitro Fermentation

The human fecal samples used in the present research were collected as previously described [14]. All individuals provided a signed consent before entering the trial. The human experiments in the present study were approved and supported by the Ethical Committee of Ocean University of China, School of Medicine and Pharmacy (Permission No. OUC-2020-1008-01). The fresh fecal samples (about 300 g) were collected into 50-mL sterile tubes and stored at −120 °C. The enterotype of the gut microbiota was determined using the method established by Liang et al. [13].

Batch fermentations of QOS, KOS, and AOS were performed in anaerobic conditions at 37 °C using Hungate tubes. An AW 500SG anaerobic chamber (Electrotek Ltd., Shipley, UK) was used to provide the anaerobic environment (80% N_2_, 10% H_2_ and 10% CO_2_). The VI medium containing QOS, KOS, or AOS at a concentration of 8 g/L was used for the fermentation, as previously described [14,25,26]. The fecal suspensions (20%, *w*/*v*) were prepared by dissolving the fresh fecal samples with sterile and pre-warmed PBS at 37 °C. For the batch fermentation, 1 mL of fecal suspension was anaerobically inoculated into 9 mL of VI growth media. All the fermentation experiments were performed in triplicates. After 48 h, the fermentation was terminated and the medium was carefully collected for further analysis.

### 4.3. Carbohydrate Utilization Analysis and SCFAs’ Analysis

Thin-layer chromatography (TLC) was performed using the method previously described [14,25,26]. Briefly, the collected fermentation medium was first centrifuged at 10,000× *g* for 5 min to remove the bacteria. After that, 0.2 μL of supernatant was loaded onto a Merck silica gel-60 TLC plate (Darmstadt, Germany) and was developed using a resolving solution containing formic acid, *n*-butanol, and water (6:4:1, *v*/*v*/*v*). The carbohydrate was visualized using the aniline-diphenylamine phosphate reagent, as previously described [14,26]. The total carbohydrate in the medium before and after fermentation was determined using the phenol-sulfuric acid method. A well-established HPLC (Agilent 1260, Santa Clara, CA, USA) method coupled with an Aminex HPX-87H ion-exclusion column (Bio-Rad, Hercules, CA, USA) was used to analyze the production of SCFAs during fermentation [14].

### 4.4. High-Throughput Sequencing and Bioinformatic Analysis

The gut bacteria were obtained from 5 mL of the final fermentation media at 48 h by centrifuging at 10,000× *g* for 5 min. The Qiagen QIAamp DNA Stool Kit (Hamburg, Germany) was applied to extract the metagenomic DNA of the bacteria. A pair of universal primers (338F and 806R) were used to specifically amplify the V3–V4 hypervariable regions of the 16S gene. The obtained amplicons were sequenced and analyzed using Illumina PE300 (San Diego, CA, USA) from Shanghai Majorbio Bio-pharm Biotechnology Co., Ltd. (Shanghai, China). The *α*-diversity, *β*-diversity, and LEfSe analyses of the gut microbiota were conducted using the online Majorbio Cloud platform (https://cloud.majorbio.com, accessed on 10 February 2020), as previously described [14].

### 4.5. Statistical Analysis

Data are expressed as mean ± SEM. Statistical analysis was performed using ANOVA with post hoc Tukey’s multiple comparisons test (GraphPad, San Diego, CA, USA). The results were considered statistically significant at *p* < 0.05; * *p* < 0.05 and ** *p* < 0.01. The LEfSe analysis was performed at the genus level. Only taxa with an LDA score >2 are listed.

## Data Availability

The data presented in this study are available on request from the corresponding author.

## Supplementary Materials

The following are available online at https://www.mdpi.com/article/10.3390/polym14040770/s1. Figure S1: Gut microbiota composition of the three human fecal samples at the genus level; Figure S2: Gut microbiota composition of the three human fecal samples at the phylum level; Figure S3: Chemical structures of QOS (A), KOS (B), and AOS (C).
