# Supplementary material for: Enterotype-Specific Effect of Human Gut Microbiota on the Fermentation of Marine Algae Oligosaccharides: A Preliminary Proof-of-Concept In Vitro Study
Source: Polymers (Basel). 2022 Feb 16;14(4):770. doi: 10.3390/polym14040770 (PMC8876871; doi:10.3390/polym14040770)
Supplement: Supplementary file 1 [file polymers-14-00770-s001.zip › polymers-1559489-supplementary.pdf]

Supplemented data

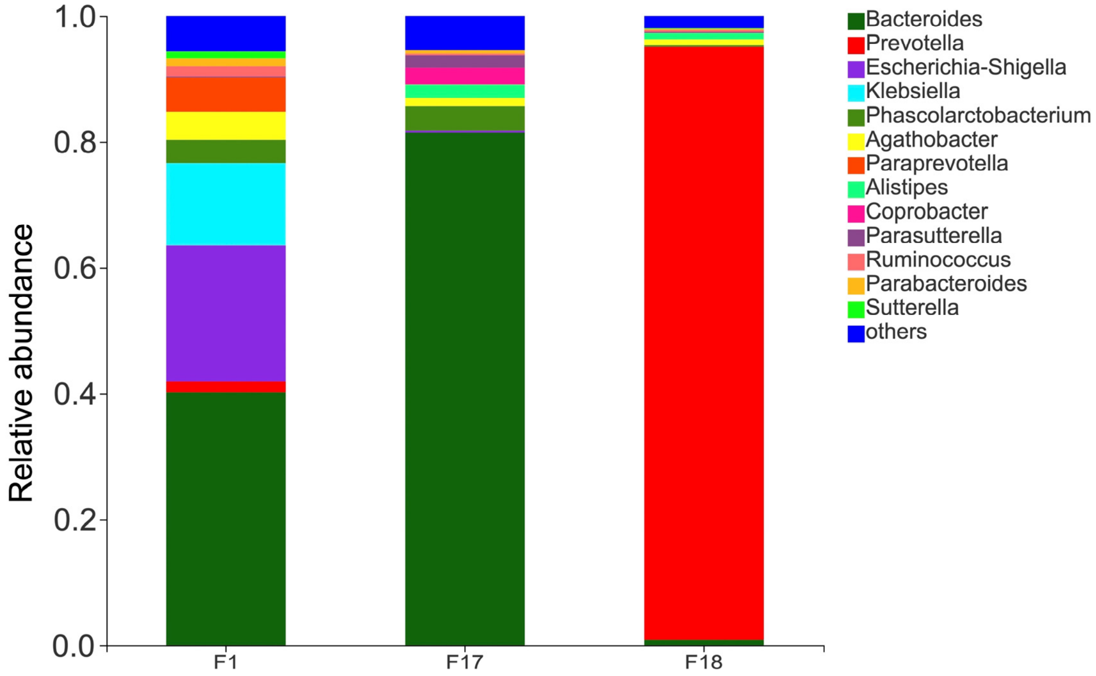

Figure S1. Gut microbiota composition of the three human fecal samples at the genus level. The samples were labeled as F1, F17 and F18. F1 was identified as an *Escherichia* enterotype microbiota. F17 was identified as a *Bacteroides* enterotype microbiota. F18 was identified as a *Prevotella* enterotype microbiota.

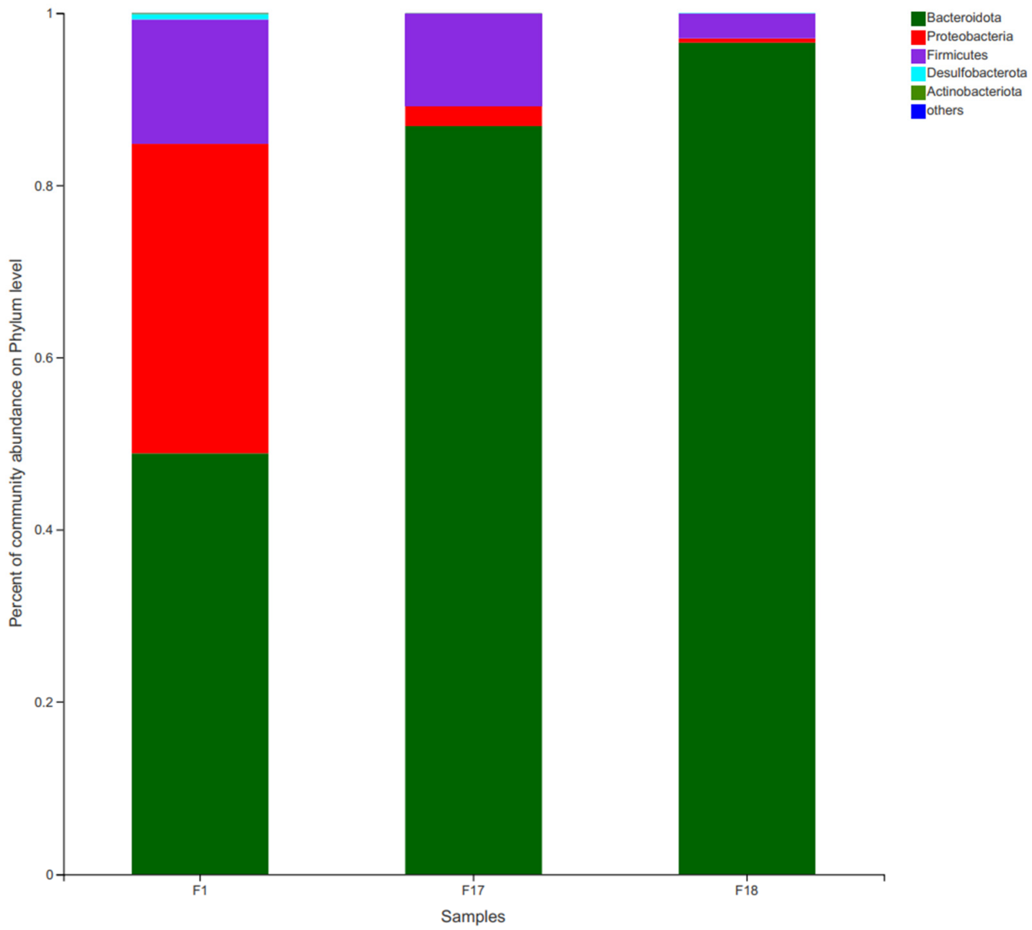

Figure S2. Gut microbiota composition of the three human fecal samples at the phylum level. The samples were labeled as F1, F17 and F18. F1 was identified as an *Escherichia* enterotype microbiota. F17 was identified as a *Bacteroides* enterotype microbiota. F18 was identified as a *Prevotella* enterotype microbiota.

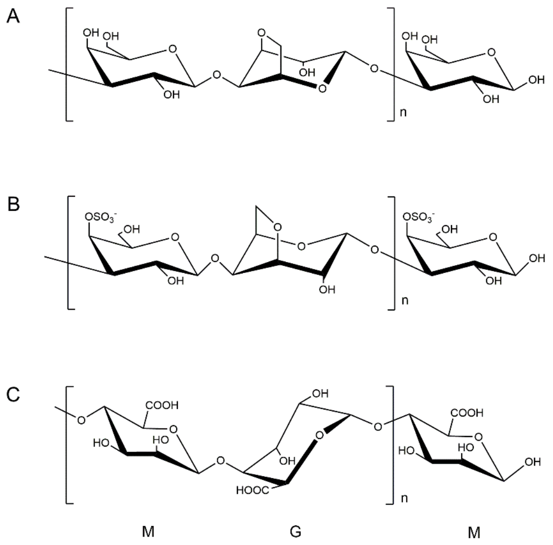

Figure S3. Chemical structures of QOS (**A**), KOS (**B**) and AOS (**C**).
